# Spatial Heterogeneity of Electrical Restitution as a Predictor of Ventricular Tachyarrhythmias: A Lumped-Parameter Approach

**DOI:** 10.1161/JAHA.112.002949

**Published:** 2012-08-24

**Authors:** Vladimir Shusterman

**Affiliations:** School of Medicine, Division of Cardiology, University of PittsburghPittsburgh, PA

**Keywords:** arrhythmia, electrocardiography, electrophysiology, ventricular tachycardia

Estimating an individual's risk for ventricular tachyarrhythmia (VTA) and sudden cardiac death (SCD) and identifying patients at high risk who would benefit the most from an implantable device remain key challenges for the cardiac electrophysiologist. A sudden onset and broad spectrum of precipitating mechanisms and triggers are the hallmarks of these life-threatening events, making them particularly difficult to foresee. Additional challenges arise because of variations in anatomy and tissue composition when cardiac electrical activity is measured at the body surface, relatively far away from its electrical sources in the heart.

## Spatial Repolarization Heterogeneity and Its Dynamics as Predictors of VTA

Despite these formidable challenges, significant progress has been made in arrhythmia risk stratification. A large body of evidence collected from theoretical, experimental, and clinical studies has shown that spatial repolarization heterogeneity can precipitate regional conduction slowing and unidirectional block and can ultimately lead to the initiation of reentrant arrhythmias.^1,2^ The proarrhythmic effects of spatial repolarization heterogeneity can be amplified or diminished by the dynamic relationships between cardiac cycle length (or, more precisely, its diastolic interval) and action potential duration (APD).^3^ The analysis of these relationships, referred to as *electrical restitution*, proved useful for understanding the mechanisms linking the dynamics of heart-rate dependent APD adaptation to arrhythmogenesis and for determining critical parameter regimes that can be proarrhythmic. In particular, the steepness of the restitution curve has been identified as one of the key factors precipitating T-wave alternans, a proarrhythmic form of repolarization instability.^3^ The analysis of theoretical properties and experimental relationships between APD restitution and arrhythmias has been the subject of intensive research in the past decade.^4^ Dynamically induced elevation of spatial repolarization heterogeneity has been shown to promote inducibility of ventricular fibrillation in experimental studies.^3^ Nevertheless, because of the complexity of restitution relationships in a real-life setting, with freely varying cycle lengths and other confounding factors, analysis of the APD restitution has not translated to the clinical practice of cardiac electrophysiology.

## A Lumped-Parameter Approach to Risk Stratification

In this issue of the *Journal of the American Heart Association* (*JAHA*), Nicolson et al^5^ present a pilot study of a single-valued composite index, referred to as the Regional Restitution Instability Index (R2I2), which incorporates both (1) an estimated spatial heterogeneity across the 12 surface ECG leads and (2) restitution-related characteristics of repolarization over a range of pacing-induced cardiac cycle lengths. The proposed lumped-parameter metric uses the location of the T wave's peak as a key fiducial point, and thus it is also influenced by the symmetry and waveform characteristics of the T wave, which can undergo changes in the presence of ischemia and repolarization heterogeneity.^6–8^

To test the accuracy of R2I2, the authors conducted a small but well-designed retrospective study that included 3 groups: (1) a Control group of 29 patients with supraventricular tachyarrhythmias, (2) a Primary Study group in which the method was refined, and (3) a Replication group in which the method was tested. The study showed that R2I2 identified the subjects who experienced VTA/SCD independently of the demographic and clinical factors included in the analysis. Moreover, using cardiac magnetic resonance imaging, the authors showed that R2I2 correlates with the extent of the peri-infarct zone. In contrast, there was no correlation between R2I2 and either QT dispersion or T_peak_ – T_end_ interval, and the latter 2 indices had no statistically significant association with the occurrence of arrhythmias in this patient population.

## Discussion

The pilot results reported by Nicolson et al^5^ show potential utility of R2I2 for predicting arrhythmic events and warrant further investigation in a larger patient population. In addition, this pilot study raises, as does virtually every novel approach, several nontrivial research questions that deserve discussion.

First, we note that R2I2 is a composite index that incorporates both spatial heterogeneity across the 12 leads and restitution-related temporal features. Combining such disparate spatial and temporal features in a single index can be useful for summarizing the most important properties of a complex, multicomponent process in a simple form. This approach is often applied in computational biology and biophysics, despite a tradeoff associated with the difficulties of post hoc analysis, to discern the effect of each participating factor included in a composite metric. In electrocardiography, combining depolarization and repolarization properties in a single index (the QRST integral) also has been used for several decades, since Wilson et al^9^ introduced it as a measure of activation-independent repolarization heterogeneity. Composite, spatio-temporal descriptors of T-wave morphology in the form of T-wave vector loop and its dispersion have proved useful for VTA/SCD risk stratification after myocardial infarction.^10^

Selecting the peak of the T wave as a key fiducial point and analyzing the relationship between the QT_peak_ (the period from S2 QRS onset to the S2 T_peak_) and T_peak_Q (period from T_peak_ on the last beat of the drive train to the S2 QRS onset) intervals as a surrogate marker of restitution have practical benefits. In most cases, the location of the T wave's peak on the surface ECG is easier to determine than the end of the T wave, which gradually merges with the isoelectric line, often obscured by noise and baseline wander.^11^ (Notable exceptions include biphasic and notched T waves, in which the location of T-wave peak may become uncertain.)

In each individual body-surface ECG lead, the location of the T-wave peak represents a combination of local (transmural) gradients and global (apex–base) effects. These locations are clustered around the mean repolarization time, which can be accurately determined by using the time of the T-wave peak on the root-mean-square curve.^12^ However, the 2-dimensional space of QT_peak_ and T_peak_Q intervals represents a significant departure from the traditional restitution relationship, expressing adaptation of the cardiac APD to changes in the diastolic interval. Indeed, the QT_peak_ interval entails both depolarization and the early part of repolarization, whereas the T_peak_Q interval includes late repolarization and the entire ventricular diastolic interval. Thus, the slope of the relationship between the 2 intervals, utilized in the R2I2 index, is equivalent to a ratio *(R*):


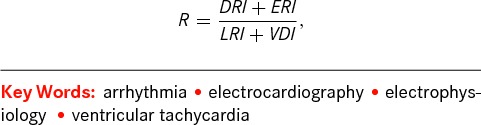


where *DRI* indicates depolarization interval; *ERI*, early repolarization interval; *LRI*, late repolarization interval; and *VDI*, ventricular diastolic interval. Given that the repolarization interval is usually longer than depolarization and that changes in depolarization time are usually smaller than those in repolarization time, the *R* ratio could be influenced significantly by changes in T-wave symmetry and waveform pattern. As mentioned earlier, changes in T-wave morphology have been linked to the risk of VTA/SCD in patients with ischemic cardiomyopathy and prior myocardial infarction.^10^ Further complicating this conundrum, an upsurge in depolarization heterogeneity reported shortly before VTA initiation in patients with heart failure may affect the *R* ratio as well.^2^ It remains to be seen how the R2I2 index is affected by depolarization and conduction aberrations and how those changes can be differentiated from enhanced repolarization heterogeneity.

Nicolson et al^5^ appropriately point out that there is room for improvement and refinement of the R2I2 approach. Perhaps most important for making this method practically useful is the replacement of cardiac pacing by noninvasive maneuvers that can modulate heart rate (eg, exercise or pharmacological stimulation of the β-adrenergic receptors). Another area for potential improvement that warrants further investigation is the incorporation of experimentally established indices of mean activation and recovery times (ie, the peaks of the *R* and T wave on the root-mean-square curve obtained from the surface ECG).^12^ Hypothetically, this could pave the way for measuring mean repolarization time and quantifying the differences between the global mean and local peaks of the T wave in individual leads. This approach also could provide a validated measure of the global activation-recovery interval.^12^ Another area for future research is the potential use of the width of the T wave, which correlates with both ventricular dispersion and ischemia.^8^

In addition, it will be important to investigate (1) stability of the R2I2 index over time in the same individual; (2) the impact of β-blockers and other medications; (3) the importance of individual differences in heart rate; and (4) the impact of changes in T-wave morphology, particularly its biphasic and notched forms, on the R2I2 behavior. It is also important to examine the effect of anatomic differences in the orientation of the electrical vector of the heart and the associated interlead differences in depolarization and repolarization vectors on the performance of the R2I2 index. Further research also is needed to identify the relationship between R2I2 and other ECG markers of arrhythmia risk, including rhythm instabilities,^13^ QT variability,^14^ T-wave alternans,^15^ and the width of the T wave, which can be modulated by ischemia.^7^ It would be particularly interesting to compare the performance of R2I2 with second central moments, which recently have been described as quantifiers of spatial heterogeneity of cardiac depolarization and repolarization in the surface ECG, and which could predict the occurrence of arrhythmic events.^2^

To summarize, the novel R2I2 index shows promise in identifying patients who are at risk for VTA/SCD. The authors are to be commended for introducing this innovative approach and conducting a small but well-designed feasibility study that included an independent test group. If the pilot results are replicated in larger studies, this approach may find a place among clinical tools for arrhythmia risk stratification. The data presented by Nicolson et al^5^ also show that inclusion of restitution properties in composite risk assessment leads to a qualitative improvement in the predictive accuracy as compared with QT dispersion, which by itself has not proved to be a consistent measure of repolarization heterogeneity or its dispersion.^12^ One can hypothesize that the lumped-parameter approach could be further extended by incorporating a more complete set of nonredundant, proarrhythmic instabilities of rhythm, depolarization, and repolarization, as described above. Adaptive approaches that use an individual's own data as a reference for personalized calibration might also contribute to the improvement of lumped-parameter risk stratification.^13^

Finding an optimal combination of nonredundant features within a simple metric to create an accurate, practically useful test for arrhythmia risk stratification remains an open challenge. Because of the complexity and broad spectrum of underlying mechanisms of arrhythmogenesis, the number of possible parameters and their combinations may become prohibitively large for manual analysis. This is a good place and a good time to start thinking about computational methods of combinatorial optimization, which could lead us to further improvements in arrhythmia risk stratification.
